# Computed Tomography Radiation Exposure among Urinary Tract Stone Patients at Tikur Anbessa Specialized Hospital: A Retrospective Study

**DOI:** 10.4314/ejhs.v32i1.6S

**Published:** 2022-10

**Authors:** Seife Teferi Dellie, Tensae Nebiyou, Daniel Admassie Tadesse, Tesfaye Kebede

**Affiliations:** Department of Radiology, College of Health Sciences, Addis Ababa University

**Keywords:** Urolithiasis, Computed Tomography dose index volume, dose length product, effective dose

## Abstract

**Background:**

National and multinational surveys indicate large variability of Computed Tomography urinary tract Stone doses. The wide use of abdominopelvic Computed Tomography in the diagnosis, raised the issue of radiation exposure. Hence, this study was conducted to assess Computed Tomography radiation exposure of urinary tract Stone Patients there by, to compare the results from established reference values and other published studies

**Methods:**

A retrospective cross-sectional was done on 100 urinary tract Stone patients who have at least one computed tomography scan as part of their follow-up or for diagnosis purposes from February 1 to May 31, 2021, at Tikur Anbesa Specialized Hospital. Data were collected using a structured questionnaire format that evaluates the number of Computed Tomography they had, scan parameters, dose indicators, and socio-demographic characteristics. Finally, the collected data were analyzed using statistical software SPSS version 22

**Results:**

Out of 100 patients 3.6%of our patients have radiation exposure of more than 4mSv, which is the standard for low-dose Computed Tomography. The median radiation exposure is 1.27mSv per scan. Exposure factors like tube current, tube current products, dose length product, and scan range all have similar values with an almost null interquartile range. All the scans that overpassed the low dose threshold(4mSv) were done outside Tikur Anbesa Specialized Hospital.

**Conclusion:**

Our study showed that Tikur Anbesa Specialized Hospital's low-dose CT protocol for patients with urinary tract Stone is well optimized as opposed to non- Tikur Anbesa Specialized Hospital.

## Introduction

Urolithiasis is the primary cause of urologic admission in Tikur Anbesa Specialized Hospital(TASH). Around 22.3% of urology patients that are admitted to TASH are due to urolithiasis (1). In the 21^st^ century, non-contrast abdominopelvic CT is becoming the imaging modality of choice in the detection of urinary calculi (2). It has proven to be a good imaging modality in the detection, characterization, and treatment planning of urolithiasis patients with remarkable sensitivity, specificity, and accuracy (2).

The wide use of this non-contrast abdominopelvic CT for detection and follow-up of patients with urolithiasis brought a concern of radiation exposure which may increase the risk of patients developing secondary malignancies, especially in the young population. Therefore, exposing patients to multiple scans of standard abdominopelvic CT for urolithiasis was inconsistent with the ALARA (as low as reasonably achievable) principle. Currently, the American college of radiology recommends the use of low-dose CT for flank pain. Despite the advocation of low dose CT for imaging of urolithiasis different institutions have different radiation dose utilization determined by scanner type or manufacturer, the protocol they utilize, tube rotation speed, helical pitch, collimation, etc (3). A meta-analysis study was done in 2008 to evaluate the performance of low-dose CT defined by an estimated effective dose of <3mSv showed that it can diagnose urolithiasis with a sensitivity of 96.6% and specificity of 95%(4).

Effective dose (ED) is an important dose quantity related to the probability of health detriment due to stochastic effects, which takes account of the relative radiosensitivity of the various organs in the scanned region. It can be utilized for comparison purposes between studies(5). A practical approach for assessing the derived dose quantity ED is to use the DLP value, displayed on the console after the examination, using age and region-specific conversion factor (k = 0.015 mSv/mGy-cm ) for a CT of abdomen/pelvis (6)which are considered independent of CT scanner type and manufacturer. Although the ICRP fails to state the dose limit for medical exposure it can be inferred from atomic bomb survivors that 20–30msv of effective dose is associated with a significantly increased risk of solid tumors (7).

In CT examinations almost all scanners today display patient dose descriptors, like CTdose index volume (CTDIvol) and dose length product(DLP). These dose descriptors don't directly measure the radiation dose that the patient absorbs but rather quantify the radiation that the patient is exposed to radiations (8). The CT dose index volume (CTDIvol) measured in mGy measures the radiation output of a CT scanner which allows for the comparison of radiation output of different CT scanners while the commonly used index dose length product(DLP) considers the length of the scanas (DLP (mGy*cm) = CTDIvol x scan length length) (9–11). The above-mentioned indexes play a significant role in understanding and improving the safety of imaging because they provide readily available and controllable measures (8).

The number of CT machines used in health institutions in Ethiopia is increasing. Similarly, the number of patients that are sent for abdominopelvic CT for diagnosis and follow-up of urolithiasis in Tikur Anbessa Specialized Hospital is increasing. Hence, the present survey aims at estimating the effective dose of patients that are sent for abdominopelvic CT for diagnosis and follow-up of urolithiasis in Tikur Anbessa Specialized Hospital.

## Methods and Material

**Study design**:The study was conducted in Tikur Anbessa Specialized Hospital (TASH), College of Health Science, Addis Ababa University, Addis Ababa, Ethiopia from February 1 to May 31, 2021. It utilizes a retrospective cross-sectional study design. The study deals with all adult patients who were greater than 18 years of age who had abdomen/pelvic CT for evaluation of flank pain hematuria and recurrent Urinary tract infection (UTI). The study also includes all adult patients on follow-up and or new patients suspected of urinary stone disease in TASH who had abdominopelvic CT for diagnosis or follow-up. TASH is chosen as a study place since the hospital has the largest number of urologists and nephrologists and the hospital is the main referral center for patients with urolithiasis. All abdominal/abdominopelvic CT scans performed at TASH during the study period and urolithiasis patients referred to TASH as part of their follow-up were the source population. All-new adult urolithiasis patients greater than 18 years of age presenting for suspected urinary stone disease in TASH or adult urolithiasis patients who are in follow-up and have at least one abdominopelvic CT scan during the study period were the study populations.

**Sample size and sampling methods**: A convenience nonprobability sampling method was used to collect data on patients who had their computed tomography scan for urolithiasis or symptoms related to urolithiasis from February 1 to May 31, 2021 until the sample size is attained. Since there are no previous studies in our country the sample size was calculated considering 95% confidence interval with 10% margin of error, 50% distribution employing single population formula to be 100.

**Data collection method**: Data were collected using a structured data collecting instrument. The data collecting format has included quantities for assessing scan parameters and CT dose indicators. The data collecting format also had sociodemographic characteristics like age and sex. The selected patients were given a phone call, where their phone number is retrieved using ‘ICARE’ an electronic health record system practiced in TASH. They were asked for consent and if they have a previous CT scan done in the last five years for urolithiasis or symptoms of urolithiasis. If their previous scan was done in TASH it will be retrieved from TASH's PACS (picture archiving and communication system). For those outside TASH they were told to bring their digital optical disc data storage given from their respective institution in their convenience.

**Data processing and analysis**: In this study, the data was filed by google forms where the data can beimmediately checked. This will typically be essential to examine the data and prevent possible data processing errors (consistency errors, implausible values, duplicating errors, transpositions, copying errors). The data is then fed to the statistical software SPSS version 22. After being further evaluated by the software for possible errors the data is processed by the same software.

All available CT scans performed within the last 5 years were retrospectively reviewed to determine the patients' maximum EDose for a single 12-month period and a total of five years. Finally, a multivariate logistic regression analysis was performed to determine the scan characteristics that significantly affect the effective dose. A p-value <0.05 was considered statistically significant

**Ethical consideration**: To respect the study group's bill of rights, the study was conducted after approval by the Research and Ethics Committee of the department, which is autonomous to give ethical clearance for research proposals below Ph.D levels. Any piece of information was kept confidential by not recording the names of the study populations.

## Results

A total of 100 patients with 109 CT scans have met the inclusion criteria and their CTs have been evaluated. Out of 100 evaluated patients, 38% were female and the rest 62% were male. The patients evaluated were with a minimum age of 18 and maximum age of 70 years and an average of 35.4 years. All the evaluated patients have noncontrast CT , and none of them had post-contrast CT or multiphasic CT. All the scanners used helical scanning mode. All scans used a constant pitch, kVp, and beam width with values of 0.98, 120, and 40mm respectively.

The median and interquartile range of the evaluated CT scan's exposure parameter and CT dose indicators values for abdominopelvic CT examination of urolithiasis patients at TASH as well NON -TASH are summarized in Table 1. It shows that the values are very constant or homogenous with no significant variation. Similarly Table 2 shows the correlation between mAS, kVp, scan range, and no of CT scan taken in determining the effective dose. Bivariate correlation using Pearson's correlation coefficient showed that mAs were statistically significant in determining the effective dose(P-value <0.05).

**Table 1:** Median and interquartile range of tube current product tube current scan range CTDIvol and dose length product of the evaluated CT scans in TASH February 1 to May 31, 2021.

	mAs median(IQR)	mA median(IQR)	Scane range median(IQR)	CTDIvol median(IQR)	DLP median(IQR)
All CT scans	18.3(18.3–18.3)	30(30–30)	41.3(46–39.4)	1.565(1.57–1.56)	95.4(90.69–80.72)
TASH	18.3(18.3–18.3)	30(30–30)	42.1(45.75–39.2)	1.565(1.57–1.56)	84.22(90.69–80.72
Non TASH	162(162–162)	270(270–270)	57.89(57.89–57.89)	10.28(10.28–10.28)	595.06(595.06–595.06)

**Table 2:** correlation between mAS, kVp, scan range, number of CT scan taken in determining the effective dose in TASH from February 1 to May 31, 2021

*CT scan parameters*		*Effective dose*

	*Pearson* *Correlation*	*Sig. (2-tailed)*
***mAs***	.998**	*<0.01*
***kVp***	*a*	*a*
***Scan range***	*0.023*	*0.818*
***No of CT taken***	*0.152*	*0.130*

a = the data is constant no correlation can be done,

** significant correlation

**Figure 1:**
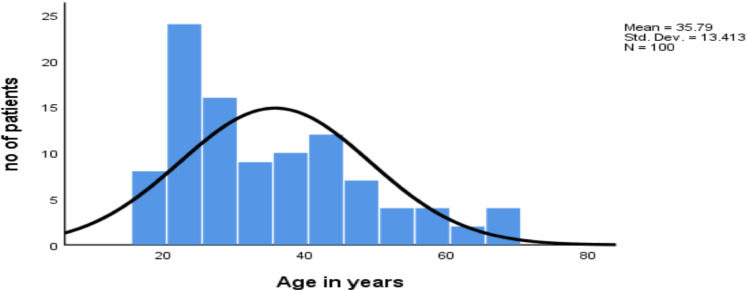
Bar graph showing patients' age distribution

**Figure 2:**
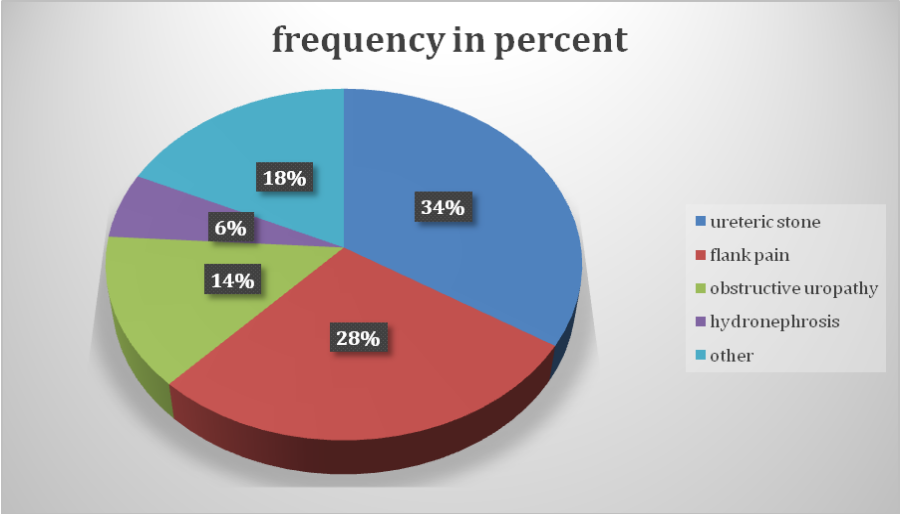
Pie chart showing common indications for the CT scans

As shown in Figure 1, most of the study participants were middle-aged patients. Out of the evaluated patients, only 9 patients(9%) have had more than one CT performed during the last 5 years. As shown in fig 2, the commonest indication for the CT scans is flank pain while the renal or ureteric stone is the second most common indication for the scans. The follow-ups for already diagnosed urolithiasis patients were the commonest indication for the second CT.

**Figure 3:**
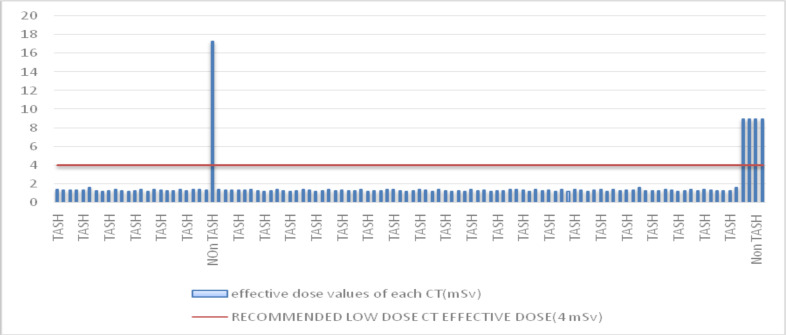
Bar graph showing each evaluated CT scan effective dose compared to the recommended low dose (4mSv) CT scan.

The effective dose of all scans has similar values except for four outliners as depicted in Figure 3. Out of the 100 patients, only 4(3.6%) patients had a calculated effective dose of >4mSv which is greater than what is considered a low dose CT. On sub-analysis, all of these CT scans were done in non-TASH institutions (**Error! Reference source not found.**. Similarly out of the 100 patients, only 4(3.6%) patients had a CT dose index >2mGy which is greater than what is considered a low dose CT and this too is from non-TASH institutions (**Error! Reference source not found.**.

## Discussion

The results of the study revealed that there is a significant variation of effective dose and exposure factors in CT scan done in TASH and Non-TASH as compared with the stated low CT dose. As opposed to the research done in (4)which considered an effective dose at or less 3 mSv and CTDI_VOL_ of 1.8mGy, in our studies, we use a higher threshold of 4mSv and CT-dose index of 2mGy as a cut-off for low dose CT scan protocols for kidney stones. This threshold is set since the urinary stone is surrounded by low-density tissue. The research done in (12)illustrated that 7.5% of scans labeled reduced dose CT examination less than an effective dose of 3mSv. In our study, we found that those patients that came from non-TASH had 3.6% of the scans have an effective dose and CTDI_VOL_ greater than 4mSv and 2mGy respectively. Compared with Non-TASH hospitals, all patients who had their scan in TASH have received radiation below the stated ‘low dose’ protocols. Even though the amount of data we have may not be sufficient to give a concrete conclusion the result indicates that scans outside TASH may be conventional CT protocol is being used. The main reasons for having a low CTDI_VOL_, DLP and effective dose with almost null interquartile range at TASH is the usage of fixed mA, mAs and kVp as indicated in table 1and table 2 According to previous studies, low-dose CT has comparable sensitivity and specificity compared to conventional CT in detecting stone (4,13)

Contrasting observations were made among various similar studies(14–16). By the research done in (14), they found that out of 389 total patients 26% and 6% had an effective dose of >20mSv and >50mSv respectively(14). Similarly, in 2009 a research done at Duke medical center in north California on radiation exposure in acute and short-term management of urolithiasis patients found that 20% of patients had radiation exposure of >50mSv in a year(17). In our study, however, none of our patients have radiation exposure >20mSv per year.

In 2006 a study on radiation dose associated with unenhanced CT for suspected renal colic in determining the incidence of repeated unenhanced CT examinations, they found that 4% had three or more CT examinations in a year and one patient even have 18 scans (18). The research in California on computed tomography radiation exposure among referred kidney stone patients showed that 25% of patients had two or more scans in 5 years (14). The research at Duke medical center in northern California on radiation exposure in the acute and short-term management of urolithiasis patients showed that patients on average had 4 examinations per year (17). In our study, only 9% of patients had two scans and none of our patients had scans more than two CT scans per year. This shows a significantly lower rate of repetition of examination compared to previous studies. The possible explanation for these is the relative scarcity of CT in our study area compared to the previous study areas where physicians may prefer to use other diagnostic methods rather than CT for evaluation of patients suspected of urolithiasis.

Our study is limited by its retrospective nature where recall bias can significantly limit our study. Secondly, our study solely focused on radiation exposure from CT but patients can be exposed to radiation through other imaging modalities like fluoroscopic procedures therefore the radiation exposure presented in our study may be underestimated. Thirdly, our study primarily focused on TASH patients and therefore is limited by lack of multi-institutionality. Lastly, the researcher does not know details of the non-TASH CT scans capacity such as slice capacity and protocol used.

Our study concluded that TASH's low-dose CT protocol for patients with urolithiasis is well optimized and patients are not being overexposed but even with the limited data we have non-TASH institutions are likely using non-optimized CT scans and patients may be a victim of radiation overexposure for either diagnosis or follow of urolithiasis. The authors, therefore, recommend a large-scale multi-institutionalized study including private and other government hospitals for the formation of standard national guidelines using scan parameters and radiation dose indicators for urolithiasis patients in Addis Ababa Ethiopia.

## Figures and Tables

**Figure 4:**
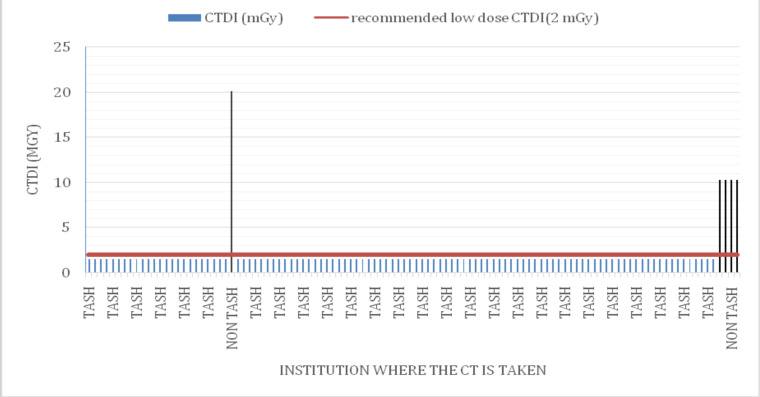
Bar graph showing CT dose index volume(CTDIvol) (measured in mGy) values of all the CT scans done in TASH and non-TASH institutions compared to the recommended low dose CTDIvol(2mGy)
